# Supplementary material for: Sweet spot in music—Is predictability preferred among persons with psychotic-like experiences or autistic traits?
Source: PLoS One. 2022 Sep 29;17(9):e0275308. doi: 10.1371/journal.pone.0275308 (PMC9521895; doi:10.1371/journal.pone.0275308)
Supplement: S1 Table — All tests are two-sided. CAPEp = the positive subscale of the Community Assessment of Psychic Experiences, AQ-short = the abridged version of the Autism Spectrum Quotient, ACE-IQ = an abbreviated version of the adverse childhood experiences international questionnaire, BAISv = the Vividness subscale of the Bucknell Auditory Imagery Scale (with some revisions), Training = years of music training, Daily listening = hours spent listening to music on a typical day. Higher mood values reflect more positive mood. Due to non-normality, Mann-Whitney tests were used to compare groups, in which the effect sizes reflect rank biserial corelations. The only exception was BAISv, where a Welch’s test (and Cohen’s d) was used due to heteroscedasticity. * Not significant using Šidák corrections, new α = .0073. (PDF) [file pone.0275308.s001.pdf]

S1 Table. Demographics and group comparisons for participants with and without Wundt curves using complexity scores.

	Wundt curve (n = 181)		No Wundt curve (n = 140)				
	Mean (*SD*)	Median (range)	Mean (*SD*)	Median (range)	t-test	*p*-value	Effect size
CAPEp	1.48 (0.32)	1.45 (2.3)	1.40 (0.27)	1.35 (1.40)	U = 10485	.008*	-.172
AQ-short	2.20 (0.38)	2.21 (2.50)	2.29 (0.42)	2.29 (2.25)	U = 14241	.057	.124
ACE-IQ	1.76 (1.58)	1.00 (6.00)	1.98 (1.70)	2.00 (6.00)	U = 13529	.288	.068
BAISv	28.87 (5.76)	29.00 (32.00)	28.59 (6.36)	29.00 (34.00)	*t*(283.3) = -0.408	.684	-.046
Mood	3.61 (0.63)	4.00 (4.00)	4.56 (0.74)	4.00 (4.00)	U = 12040.5	.390	-.050
Training	5.00 (6.85)	3.00 (43.00)	6.57 (8.41)	3.00 (49.00)	U = 13689	.208	.080
Daily listening	2.20 (1.28)	2.50 (4.50)	1.94 (1.18)	1.50 (4.00)	U = 11171	.061	-.118

All tests are two-sided. CAPEp = the positive subscale of the Community Assessment of Psychic Experiences, AQ-short = the abridged version of the Autism Spectrum Quotient, ACE-IQ = an abbreviated version of the adverse childhood experiences international questionnaire, BAISv = the Vividness subscale of the Bucknell Auditory Imagery Scale (with some revisions), Training = years of music training, Daily listening = hours spent listening to music on a typical day. Higher mood values reflect more positive mood. Due to non-normality, Mann-Whitney tests were used to compare groups, in which the effect sizes reflect rank biserial corelations. The only exception was BAISv, where a Welch's test (and Cohen's d) was used due to heteroscedasticity.

* Not significant using Šidák corrections, new α = .0073.
