# Supplementary material for: Sweet spot in music—Is predictability preferred among persons with psychotic-like experiences or autistic traits?
Source: PLoS One. 2022 Sep 29;17(9):e0275308. doi: 10.1371/journal.pone.0275308 (PMC9521895; doi:10.1371/journal.pone.0275308)
Supplement: S2 Table — All tests are two-sided. Complexity = preferred complexity indicated by peaks, Entropy 50 ms = preferred entropy (indicated by peaks) calculated with 50 ms time windows, Entropy 20 ms = preferred entropy (indicated by peaks) calculated with 20 ms time windows, CAPEp = the positive subscale of the Community Assessment of Psychic Experiences, AQ-short = the abridged version of the Autism Spectrum Quotient, ACE-IQ = an abbreviated version of the adverse childhood experiences international questionnaire, BAISv = the Vividness subscale of the Bucknell Auditory Imagery Scale (with some revisions), Training = years of music training, Daily listening = hours spent listening to music on a typical day. Higher mood values reflect more positive mood. * Not significant using Šidák corrections, new α = .0011. (PDF) [file pone.0275308.s002.pdf]

S2 Table. Kendall's rank correlations.

	Complexity (n = 181)		Entropy 50 ms (n = 183)		Entropy 20 ms (n = 183)	
	τ	*p*-value	τ	*p*-value	τ	*p*-value
CAPEp subscales	.048	.362	.111	.032*	.119	.023*
Paranoia	.036	.509	.096	.075	.093	.085
Bizarre experiences	.076	.162	.122	.024*	.131	.016*
Grandiosity	.001	.981	.058	.295	.055	.328
Magical thinking	-.068	.234	.042	.464	.038	.502
Hallucinations	.048	.424	.071	.233	.102	.088
AQ-short subscales	.050	.330	.054	.286	.048	.345
Social skills	.017	.747	-.019	.721	-.037	.482
Routine	-.006	.908	-.075	.161	-.047	.383
Switching	.082	.128	.040	.447	0.23	.664
Imagination	.004	.939	.039	.460	.040	.438
Numbers and Patterns	.064	.223	.124	.018*	.135	.010*
ACE-QI	.044	.426	.025	.651	.044	.417
BAISv	.074	.157	.074	.150	.049	.342
Mood	-.081	.181	.104	.076	.090	.125
Training	.006	.913	.004	.938	-.002	.972
Daily listening	.041	.466	.058	.299	.055	.325

All tests are two-sided. Complexity = preferred complexity indicated by peaks, Entropy 50 ms = preferred entropy (indicated by peaks) calculated with 50 ms time windows, Entropy 20 ms = preferred entropy (indicated by peaks) calculated with 20 ms time windows, CAPEp = the positive subscale of the Community Assessment of Psychic Experiences, AQ-short = the abridged version of the Autism Spectrum Quotient, ACE-IQ = an abbreviated version of the adverse childhood experiences international questionnaire, BAISv = the Vividness subscale of the Bucknell Auditory Imagery Scale (with some revisions), Training = years of music training, Daily listening = hours spent listening to music on a typical day. Higher mood values reflect more positive mood.

* Not significant using Šidák corrections, new α = .0011
