# Supplementary material for: Sweet spot in music—Is predictability preferred among persons with psychotic-like experiences or autistic traits?
Source: PLoS One. 2022 Sep 29;17(9):e0275308. doi: 10.1371/journal.pone.0275308 (PMC9521895; doi:10.1371/journal.pone.0275308)
Supplement: S1 Text — (PDF) [file pone.0275308.s003.pdf]

S1 Text. Wundt effect at group level in the total sample (*n* = 321)

As in the main analysis, the linear mixed model with the total sample included participants as random intercepts. The results from the linear mixed model showed a Wundt effect between liking ratings and complexity scores in the total sample (n = 321), with a significant negative quadratic effect (β = -.17, p = .010). The linear term was not significant (β = .65, p = .399).

When replacing complexity scores with entropy scores calculated using 50 ms time windows, the linear mixed model had a significant positive linear effect (β = 64.71, p = .024) and a significant negative quadratic effect (β = -360.02, p = .001). For entropy calculated using 20 ms time windows, the model showed a significant negative quadratic effect (β = -276.72, p = .010), but the linear effect was non-significant (β = 45.41, p = .105).
