# Supplementary material for: Sweet spot in music—Is predictability preferred among persons with psychotic-like experiences or autistic traits?
Source: PLoS One. 2022 Sep 29;17(9):e0275308. doi: 10.1371/journal.pone.0275308 (PMC9521895; doi:10.1371/journal.pone.0275308)
Supplement: S2 Text — (PDF) [file pone.0275308.s004.pdf]

S2 Text. Wiener entropy

An alternative to the complexity scores was the excerpts' noisiness or uniformity (of the power spectra), known as Wiener entropy or spectral flatness. Entropy is measured on a zero to one scale, where clear and distinguishable tones lie close to zero and white noise lies close to one. We measured spectral flatness by using short-time Fourier transform. That is, we divided the audio into smaller time segments and analysed the frequency spectrum (by Fast Fourier Transform) of each segment. Frequency resolution was prioritised over time resolution, as the duration of the excerpts would ensure sufficient sound information over time. We therefore opted for longer time windows, and included two window lengths to evaluate the robustness of the analysis. The time windows were set to either 50 ms or 20 ms, with a 50% overlap, producing two entropy scores for each excerpt.

The screening procedure from the main analysis (using complexity scores) was repeated for the quadratic regressions between music liking and entropy. Only participants with quadratic components smaller than -0.1 were included, as this indicated an inverted U-shaped relationship between music liking and entropy. This resulted in a sample size of n = 183 for both 50 and 20 ms (although these samples were not identical). The same analyses from the main study were performed by replacing complexity scores with entropy scores. As in the main analysis, the linear mixed model included participants as random intercepts.

Results

The results from the linear mixed model confirmed a Wundt effect between liking and entropy scores (50 ms), with a significant positive linear effect (β = 351.61, $p < .001$) and a significant negative quadratic effect (β = -1531.05, $p < .001$). These results were replicated when using entropy scores calculated using 20 ms time windows, including both a

significant positive linear effect (β = 322.21, p < .001) and a significant negative quadratic effect (β = -1371.88, p < .001).

The partial correlation between participants' AQ-short scores and entropy (50 ms), while controlling for ACE-IQ scores and mood, was non-significant, Kendall's τ = .077, p = .938. Using entropy scores calculated with 20 ms time windows replicated these results, Kendall's τ = .074, p = .930.

The partial correlation between participants' CAPEp scores and entropy (50 ms), while controlling for ACE-IQ scores and mood, was non-significant, Kendall's τ = .100, p = .977. Using entropy calculated with 20 ms time windows replicated these results, Kendall's τ = .104, p = .981.
