# Supplementary material for: Sweet spot in music—Is predictability preferred among persons with psychotic-like experiences or autistic traits?
Source: PLoS One. 2022 Sep 29;17(9):e0275308. doi: 10.1371/journal.pone.0275308 (PMC9521895; doi:10.1371/journal.pone.0275308)
Supplement: S3 Text — (PDF) [file pone.0275308.s005.pdf]

S3 Text. Bounded maxima

We investigated whether the results were robust when the sample was not limited to those with Wundt curves. We performed quadratic regression analyses between the music excerpts' complexity scores and preference ratings for each participant, as well as between entropy scores (both 20 ms and 50 ms) and preference ratings. For participants with Wundt curves, the maxima were the peaks of the inverted U-shaped curves (as in the confirmatory analyses). For participants with positive quadratic components, where the relationship between complexity/entropy and liking was U-shaped, the preferred level of complexity/entropy corresponded to the bounded maximum of the parabola (see Fig 2, right panel). Adding participants with positive quadratic components meant that a greater portion of the sample consisted of participants who preferred either the lowest or highest levels (see S1 Fig for comparisons).

As one can argue that all except those with zero slopes will have a maximum, we excluded those with slopes close to zero. For complexity scores, 22 participants were excluded for having quadratic components larger than -0.1 and smaller than 0.1, and for not having a linear slope distinguishable from 0 (per our pre-registered criteria). No participants had quadratic components larger than -0.1 and smaller than 0.1 for entropy scores. This resulted in an increase in sample size from n =181 to n = 299 for complexity scores (top panels in S1 Fig), and from n = 183 to n = 321 for both entropy scores with 20 ms (centre panels in S1 Fig) and 50 ms (bottom panels in S1 Fig) time windows. Partial correlation from the main analyses were repeated for the complexity scores (n = 299) and entropy scores (both n = 321) using the samples with the bounded maxima. No correlations were significant. Results are presented in Table A.

Table A. Partial Kendall's rank correlations.

	Complexity (n = 299)		Entropy 20 ms (n = 321)		Entropy 50 ms (n = 321)	
	Kendall's τ	p-value	Kendall's τ	p-value	Kendall's τ	p-value
CAPEp	.041	.286	.051	.175	.043	.255
AQ-short	.031	.423	.036	.333	.034	.362

All tests are two-sided, and controlled variables were mood and sum cores from the abbreviated version of the adverse childhood experiences international questionnaire. CAPEp = the positive subscale of the Community Assessment of Psychic Experiences, AQ-short = the abridged version of the Autism Spectrum Quotient.
