# Supplementary figures and images for: Sweet spot in music—Is predictability preferred among persons with psychotic-like experiences or autistic traits?
Source: PLoS One. 2022 Sep 29;17(9):e0275308. doi: 10.1371/journal.pone.0275308 (PMC9521895; doi:10.1371/journal.pone.0275308)

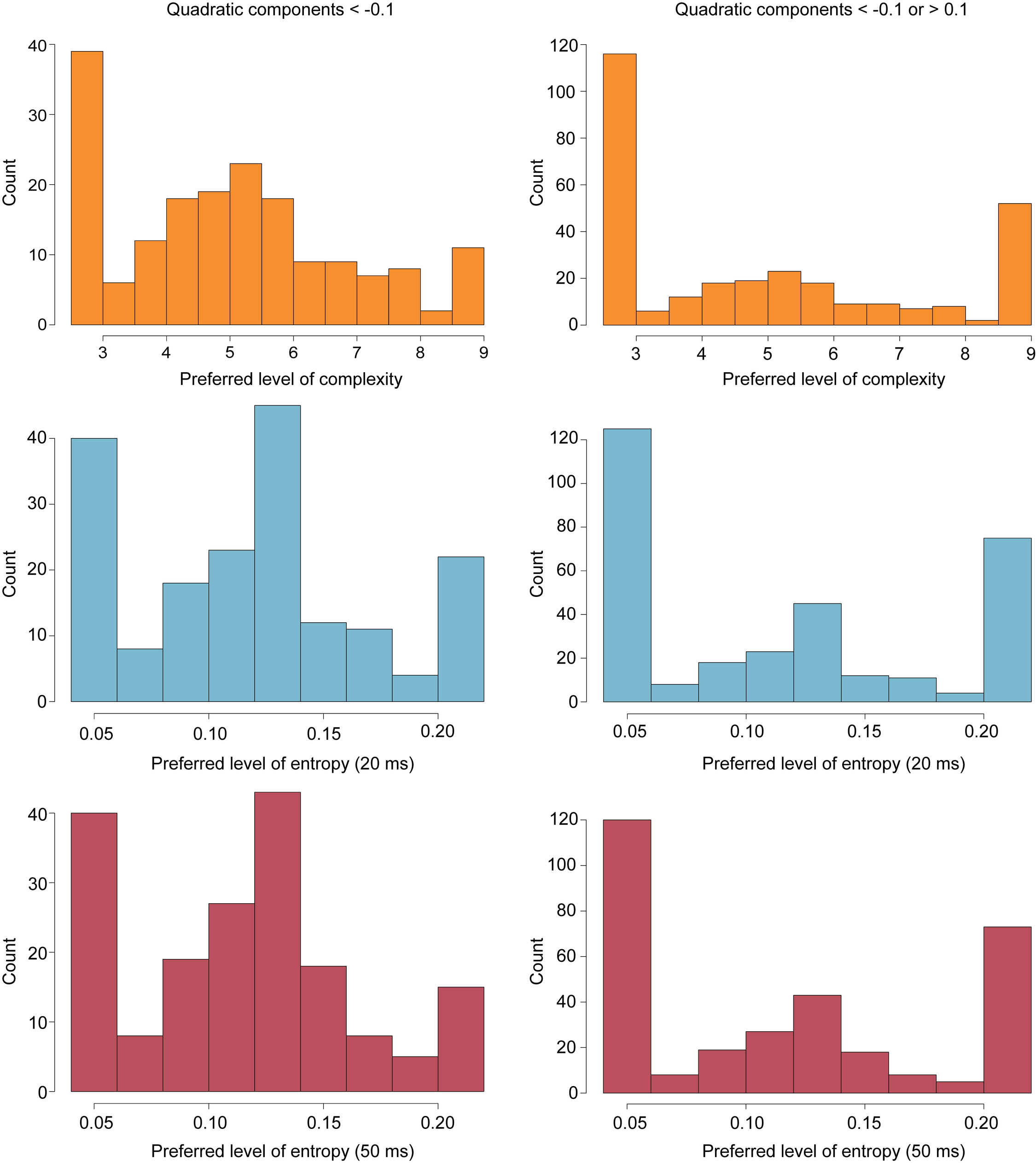

Supplement: S1 Fig — The top left panel shows the preferred complexity levels of participants with quadratic components below -0.1 (n = 181), while the top right panel consists of those with quadratic components below -0.1 and above 0.1 (n = 299). The centre left panel show the preferred entropy levels (20 ms) of participants with quadratic components below -0.1 (n = 183), while the centre right panel consists of those with quadratic components below -0.1 and above 0.1 (n = 321). The bottom left panel shows the preferred entropy levels (50 ms) for participants with quadratic components below -0.1 (n = 183), while the bottom right panel consists of those with quadratic components below -0.1 and above 0.1 (n = 321). (TIF) [file pone.0275308.s006.tif]
