# Supplementary material for: Sweet spot in music—Is predictability preferred among persons with psychotic-like experiences or autistic traits?
Source: PLoS One. 2022 Sep 29;17(9):e0275308. doi: 10.1371/journal.pone.0275308 (PMC9521895; doi:10.1371/journal.pone.0275308)
Supplement: Attachment — Submitted filename: Response to Reviewers.docx [file pone.0275308.s008.docx]

Dr. Tom Verguts

Academic Editor

PLOS ONE

Subject: Submission of revised paper [PONE-D-21-22799R1] - [EMID:a16eb5444f22855b]

Dear Dr. Verguts,

We wish to thank you and the reviewers for taking the time to read our manuscript and for giving us valuable feedback. As it seems there was only one comment, I reply to it here.

*There are two very clear suggestions that can be made based on your study: 1) make the range of complexity (much) larger; 2) include subject-specific complexity measures. Regarding 2, predictability can be varied either by changing the items or by changing the subjects (i.e., increased expertise with specific musical pieces). Indeed, predictability is an inherently subjective (in the sense of subject-specific) measure. You mention both factors briefly in your Limits and Strengths section, but I think it's worthwhile to be (even) more specific that this is the way to go for future study on this topic.*

**Reply**: We thank you for this suggested improvement. We have added a new section (“directions for future research”, lines 459-485) where we have discussed these two points in more details, as well as made recommendations for future research. We hope that our modifications have increased the standard of the manuscript sufficiently.

Kind regards,

Rebekka S. Lisøy

*Ph.D candidate*

*Norwegian university of science and technology*

*Trondheim, Norway*
